# Postnatal Growth Assessment and Prediction of Neurodevelopment and Long-Term Growth in Very Low Birth Weight Infants: A Nationwide Cohort Study in Korea

**DOI:** 10.3390/jcm13102930

**Published:** 2024-05-16

**Authors:** Min Soo Kim, Ji Won Koh, Jeongmin Shin, Sae Yun Kim

**Affiliations:** Department of Pediatrics, Seoul St. Mary’s Hospital, College of Medicine, The Catholic University of Korea, 222 Banpo-daero, Seocho-gu, Seoul 06591, Republic of Korea; kms9057@gmail.com (M.S.K.); gira610@hanmail.net (J.W.K.); emmyshin@gmail.com (J.S.)

**Keywords:** very low birth weight, extrauterine growth restriction, Fenton, Intergrowth-21^ST^, neurodevelopmental impairment

## Abstract

**Background/Objectives**: Extrauterine growth restriction (EUGR) is associated with high mortality and an increased incidence of poor neurodevelopmental outcomes in preterm infants. In this study, we aimed to compare the Intergrowth-21^ST^ (IG-21^ST^) and Fenton charts in predicting long-term neurodevelopmental and anthropometric outcomes of very low birth weight (VLBW) infants. **Methods**: Data were collected from 2649 VLBW infants registered in the Korean Neonatal Network born between 24^0/7^ and 31^6/7^ weeks of gestational age from January 2013 to December 2017. Follow-up assessments were conducted at 18–24 months of age, corrected for prematurity. Multiple logistic regression analysis was performed to evaluate the association between EUGR and long-term outcomes. **Results**: Among the 2649 VLBW infants, 60.0% (1606/2649) and 36.9% (977/2649) were diagnosed as having EUGR defined by the Fenton chart (EUGR_F_) and by the IG-21^ST^ chart (EUGR_IG_), respectively. The EUGR_IG_ group exhibited a higher proportion of infants with cerebral palsy, neurodevelopmental impairment (NDI), and growth failure. In multiple logistic regression analysis, adjusted for risk factors for long-term outcome, the EUGR_IG_ group showed higher risk of cerebral palsy (adjusted odds ratio [aOR], 1.66; 95% confidence interval [CI], 1.04–2.65), NDI (aOR, 2.09; 95% CI, 1.71–2.55), and growth failure (aOR, 1.57; 95% CI, 1.16–2.13). Infants with EUGR_F_ tended to develop NDI (aOR, 1.29; 95%CI, 1.03–1.63) and experience growth failure (aOR, 2.44; 95% CI, 1.77–3.40). **Conclusions**: The IG-21^ST^ chart demonstrated a more effective prediction of long-term neurodevelopmental outcomes, whereas the Fenton chart may be more suitable for predicting growth failure at 18–24 months.

## 1. Introduction

Despite improvements in the survival rate of preterm infants, those born very preterm still encounter an increased risk of adverse neurodevelopmental outcomes [1,2]. Extrauterine growth restriction (EUGR) poses a universal problem in preterm infants and has been associated with elevated mortality rates and an increased incidence of unfavorable neurodevelopmental outcomes [3,4]. Postnatal catch-up growth is a favorable predictor of cognitive function in term-born adolescents [5], recognizing the importance of optimal postnatal growth in determining later growth and neurodevelopmental outcomes [6].

Of the multiple factors associated with development of EUGR, birth weight is a very important contributor: very low birth weight (VLBW, birth weight [BW] < 1500 g) infants are at a higher risk of EUGR [7]. Therefore, assessing and monitoring the optimal growth of VLBW infants is essential in neonatal intensive care. The goal of identifying EUGR is to address nutritional needs and optimize growth to prevent adverse neurodevelopmental outcomes. Additionally, overdiagnosis of EUGR should be avoided, as overfeeding preterm infants in the neonatal intensive care unit (NICU) can lead to obesity and cardiovascular disease later in life [8]. Therefore, selecting an appropriate growth chart with adequate nutritional guidelines is important for identifying malnourished infants.

In practice, two growth standards are commonly used to monitor the postnatal growth of preterm infants. The Fenton preterm growth chart, revised in 2013, provides estimated fetal weight charts based on intrauterine growth and has been widely used as a reference [9]. However, the Fenton chart is based on size-at-birth measurements, which can result in differences between actual postnatal growth and the growth indicated by the curve. More recently, the Preterm Postnatal Follow-up Study (PPFS) has established standards for postnatal growth in preterm infants [10]. The Intergrowth-21^ST^ (IG-21^ST^) charts were developed from serial anthropometric measurements of preterm infants selected from the PPFS. The IG-21^ST^ charts are based on a longitudinal collection of preterm postnatal anthropometric data [10,11]. A recent nationwide cohort study in South Korea demonstrated that EUGR is more prevalent based on the Fenton chart (EUGR_F_) compared with the IG-21^ST^ chart (EUGR_IG_), and EUGR_IG_ is associated with worse clinical outcomes [12]. Additionally, a single-center retrospective study in the US suggests that EUGR_IG_ may have a stronger association with poor neurodevelopmental outcomes than EUGR_F_ [13]. Because of conceptual and methodological limitations and lack of studies on the efficacy of predicting long-term outcomes, determining the most appropriate growth chart remains a controversial issue for clinicians. Therefore, the current study aimed to compare the prevalence of EUGR defined by Fenton and IG-21^ST^ charts and to investigate the predictive power of both charts in neonatal long-term outcomes. 

## 2. Materials and Methods

The Korean Neonatal Network (KNN) is a nationwide prospective registry of VLBW infants born in South Korea. The network encompassed 77 participating NICUs and covered more than 70% of all births of VLBW infants in South Korea [14]. Each participating hospital’s institutional review board (IRB) approved data collection for the KNN, and all data were regularly monitored by the KNN data management committee. All methods were performed in accordance with relevant guidelines and regulations.

### 2.1. Study Design and Data Collection

**Figure 1:**
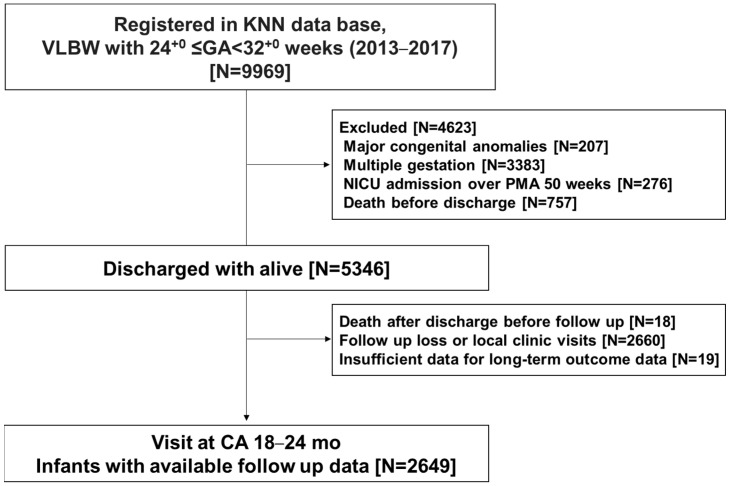
Study population flow chart. Abbreviations: CA, corrected age; GA, gestational age; KNN, Korean Neonatal Network; NICU, neonatal intensive care unit; PMA, postmenstrual age; VLBW, very low birth weight.

Among the registered population, infants with a gestational age (GA) between 24^0/7^ and 31^6/7^ weeks who were born in participating hospitals from 1 January 2013 to 31 December 2017 were eligible for inclusion. Moreover, infants who survived until NICU discharge and were followed up until 18–24 months of corrected age (CA) for prematurity at affiliated outpatient clinics were selected from the KNN registry for long-term outcome evaluation (Figure 1).

Maternal characteristics included maternal age, maternal hypertension, mode of delivery, histological chorioamnionitis, and antenatal steroid use. Neonatal variables encompassed GA at birth, BW, weight *z*-score at birth and discharge, 5-min Apgar score, and the sex of the infants. “Small for gestational age” (SGA) was defined as BW under the 10th percentile for GA and sex, according to each growth chart. The evaluated short-term neonatal outcomes included respiratory distress syndrome (RDS), treatment for patent ductus arteriosus (PDA), moderate to severe bronchopulmonary dysplasia (BPD)) [15], and necrotizing enterocolitis (NEC) ≥ stage II [16]. Severe brain injury was defined as an intraventricular hemorrhage exceeding grade 3 [17] or periventricular leukomalacia. Other outcome variables assessed included retinopathy of prematurity (ROP) [18], culture-proven sepsis in infants with clinical signs of infection, duration of parenteral nutrition, and length of NICU stay.

### 2.2. Definitions of EUGR

Gradients of z-scores for BW and weight at discharge were calculated using both the Fenton and IG-21^ST^ charts (https://intergrowth21.tghn.org/standards-tools/, accessed on 4 February 2022) [9,19,20]. We defined decreased z-scores of weight >1 at discharge compared with the z-scores of weight at birth using the Fenton chart as EUGR_F_ and the IG-21^ST^ chart as EUGR_IG_.

### 2.3. Long-Term Outcomes

The KNN follow-up protocol involved routine evaluations at 18–24 months CA for surviving infants at each participating hospital. All infants were asked to proactively visit the follow-up clinic and undergo a comprehensive assessment of growth and development. The evaluation utilized the Bayley scales of infant development, second edition (BSID-II); Bayley scales of infant and toddler development, third edition (BSID-III); and Korean developmental screening test (K-DST) for infants and children. Neurodevelopmental impairment (NDI) was defined based on meeting at least one of the following criteria: (1) BSID-II mental development index and/or BSID-II psychomotor development index score < 70; (2) BSID-III cognitive composite score, language composite score, and/or motor composite score < 85; and (3) K-DST score below -2 standard deviations, indicating NDI. Cerebral palsy (CP) was defined as the degree of functional impairment classified using the gross motor function classification system ≥ 2. In this study, growth failure was defined as a body weight measurement below the 10th percentile at follow-up assessment, following the World Health Organization child growth standard.

### 2.4. Statistical Analysis

Maternal and infant characteristics and neonatal outcomes were compared between the EUGR and non-EUGR groups using the Pearson chi-square test or Fisher’s exact test for categorical variables and Student’s *t*-test or the Mann–Whitney *U* test, as appropriate. Logistic regression analyses were performed to assess the association between EUGR and long-term outcomes. All variables found to be statistically significant in the univariate models were included in the analysis as potential confounders. Because of the multicollinearity between BW and GA, only GA was selected as a confounder. Furthermore, the sensitivity, specificity, positive predictive value, negative predictive value, accuracy, and diagnostic odds ratio (DOR) of the EUGR_IG_ and EUGR_F_ were calculated for the long-term outcomes. Continuous variables are presented as medians and interquartile ranges (IQR), and categorical variables are expressed as frequencies and percentages. Statistical analyses were conducted using R software package version 4.3.0 (R Foundation for Statistical Computing, Vienna, Austria) and SAS version 9.4 (SAS Institute, Cary, NC, USA). All tests were two-tailed, and *p* < 0.05 was considered significant.

### 2.5. Ethics Statement

The study protocol was reviewed and approved by the IRB of Yeouido St. Mary’s Hospital (IRB no. SC21ZIDE0176) and the KNN Ethics Committee (2021-057). Data were prospectively entered into the KNN registry after obtaining written informed consent from parents.

## 3. Results

### 3.1. Clinical Characteristics of Study Population

A total of 9969 VLBW infants were registered in the KNN registry during the study period. Of these, 4623 infants were excluded for the following reasons: congenital anomalies (*n* = 207), multiple gestations (*n* = 3383), not discharged until postmenstrual age 50 weeks (*n* = 276), and death before discharge (*n* = 757). Among the 5346 infants, 2697 were excluded after discharge due to post-discharge death (*n* = 18), loss to follow-up (*n* = 2660), and insufficient follow-up data (*n* = 19). Finally, 2649 infants were included in the analyses, and their information was available for the CA of 18–24 months visit (Figure 1).

Characteristics of the study population are summarized in Table 1. Based on the Fenton chart, EUGR was more prevalent: 1606/2649 (60.6%) infants were diagnosed as defined by EUGR_F_ and 977/2649 (36.9%) as defined by EUGR_IG_. Significant differences were observed in maternal and neonatal characteristics across EUGR definitions. In both classifications, a higher proportion of infants with maternal hypertensive disorders was found in the non-EUGR group, whereas maternal chorioamnionitis was more prevalent in the EUGR group. Differences in the mode of delivery were significant only according to the IG-21^ST^ definition. Infants with EUGR were born at an earlier GA than non-EUGR infants in both charts. The median (IQR) GA at birth was 27.3 (26.0–28.7) weeks for infants with EUGR_IG_ and 29.0 (27.6–30.1) weeks for non-EUGR_IG_ infants. In the case of the Fenton definition, the median GA at birth was 27.9 (26.4–29.4) weeks for infants with EUGR_F_, and 29.1 (27.7–30.3) weeks for non-EUGR_F_ infants. In both classifications, the median (IQR) BW of infants in the EUGR group was lower than that in the non-EUGR group: 1030 g (850–1240) for EUGR_F_, 1140 g (940–1300) for non-EUGR_F_, 1000 g (840–1210) for EUGR_IG_, and 1120 g (900–1300) for non-EUGR_IG_. According to the IG-21^ST^ definition, a higher proportion of male infants was observed in the EUGR_IG_ group than in the non-EUGR_IG_ group; however, no significant differences were noted in the Fenton classification. When each chart was used to evaluate SGA at birth, the proportion of infants with SGA was not significantly different between the EUGR_F_ and non-EUGR_F_ groups. However, the proportion of SGA infants was significantly higher in the non-EUGR_IG_ group than in the EUGR_IG_ group according to both the Fenton and IG-21^ST^ criteria.

According to the IG-21^ST^ classification, moderate to severe BPD was more frequent in EUGR_IG_ infants compared with non-EUGR_IG_ infants; however, no significant differences were found between the EUGR_F_ and non-EUGR_F_ groups. Except for moderate to severe BPD, other neonatal morbidities, including RDS, treated PDA, NEC, severe brain injury, ROP, and sepsis, occurred more frequently in the EUGR group than in the non-EUGR group in both charts. In both classifications, infants with EUGR required a longer duration of parenteral nutrition and experienced longer hospitalization periods than non-EUGR infants.

### 3.2. Association between EUGR and Long-Term Outcomes

All significant factors identified in the univariate analysis were included in the multivariate logistic regression model as confounding factors (Table A1). EUGR_IG_ was significantly associated with CP (adjusted odds ratio [aOR], 1.66; 95% confidence interval [CI], 1.04–2.65), NDI (aOR, 2.09; 95% CI, 1.71–2.55), and growth failure (aOR, 1.57; 95% CI, 1.16–2.13). EUGR_F_ showed a significant association with NDI (aOR, 1.29; 95% CI, 1.03–1.63) and growth failure at 18–24 months of age (aOR, 2.44; 95% CI, 1.77–3.40) (Table 2).

### 3.3. Diagnostic Effectiveness of EUGR_F_ and EUGR_IG_

To diagnose long-term outcomes, the EUGR_F_ showed a higher sensitivity than the EUGR_IG_; however, the specificities were higher in the EUGR_IG_ than in the EUGR_F_. The DORs for CP, NDI, and growth failure were greater than 1 for EUGR_IG_ and EUGR_F_. For CP, the EUGR_IG_ had a higher DOR than the EUGR_F_ (1.73 vs. 1.40, respectively). Similarly, for NDI, the DOR of the EUGR_IG_ was higher than that of the EUGR_F_ (2.08 vs. 1.59, respectively). Conversely, for growth failure, the DOR of the EUGR_IG_ was lower than that of the EUGR_F_ (1.77 vs. 2.46, respectively) (Table 3).

## 4. Discussion

To date, this is the first study to analyze two growth standards to evaluate EUGR and long-term outcome using large nationwide population. This study makes a significant contribution to the literature, as postnatal growth plays an important role in determining long-term outcomes for preterm infants. It emphasizes the need to properly utilize tools to evaluate postnatal growth. Our study has several notable findings. First, EUGR was more prevalent based on the Fenton chart. Second, EUGR_IG_ was a better predictor than EUGR_F_ for CP or NDI, and EUGR_F_ was a better predictor compared with EUGR_IG_ for growth failure at 18–24 months of CA.

### 4.1. Difference between EUGR_F_ and EUGR_IG_

EUGR is a common phenomenon in preterm infants during their NICU stay. The Vermont Oxford Network study reported that EUGR, defined as weight below the 10th percentile according to the Fenton chart, occurred in half of VLBW infants [21]. Similar to our study, EUGR defined by dynamic changes in z-scores is a widely accepted criterion. A study using the Fenton chart reported the prevalence of EUGR during NICU admission in preterm infants born at a GA of 22–32 weeks as 38–47% [22,23]. In our study population, the incidence of EUGR_F_ and EUGR_IG_ were 60.6% (1606/2649) and 36.9% (977/2649), respectively. These results are in line with previous studies comparing various growth charts; EUGR for weight was less prevalent with the IG-21^ST^ charts compared with the Fenton chart [12,24,25,26]. This difference may be expected because the Fenton chart might overestimate anthropometric data compared with the IG-21^ST^ chart. The Fenton chart is based on size-at-birth measurements for preterm infants, whereas the IG-21^ST^ chart is based on actual postnatal growth in preterm infants who experience a different environment and metabolic responses than fetuses in the intrauterine environment. As a result, more infants were diagnosed by EUGR_F_ than by EUGR_IG_.

### 4.2. Factors Associated with EUGR

In the current study, the proportion of infants with a maternal historical chorioamnionitis was significantly higher in the EUGR group than in the non-EUGR group, according to both definitions. In preterm infants, antenatal exposure to chorioamnionitis is associated with an increased risk of in-hospital morbidities [27]. These morbidities might represent conditions that disrupt nutrition potentially affecting postnatal growth. The results of our study, which assessed neonatal outcomes, also support this hypothesis. Infants in the both EUGR groups showed a significantly higher incidence of neonatal morbidities.

The most important factors related to EUGR are prematurity [28,29], and enteral nutritional support [30]. We also found that infants in the both EUGR groups were not only more immature but also had lower BWs than those in the non-EUGR. The growth of preterm infants is a developmental process; feeding intolerance, and/or difficulties in digesting decrease as infants mature [31].

### 4.3. Efficient Prediction for Long-Term Outcomes

Analyses of long-term outcomes are required to evaluate the appropriateness of growth charts in defining EUGR. In a retrospective cohort study in Taiwan, Chien et al. reported that EUGR is significantly associated with cognitive function at 24 months of CA for prematurity [32]. Zozaya et al. found that a low-weight z-score is associated with poor BSID score at 24 months of CA for prematurity in an Italian preterm cohort [33]. Our findings revealed a higher incidence of infants diagnosed with CP, NDI, and growth failure in the EUGR group at 18–24 months CA compared with the non-EUGR group, based on both definitions. Interestingly, the differences were observed between the growth charts in predicting long-term outcomes. After adjusting for confounding factors, infants diagnosed by EUGR_IG_ at the newborn period had a 1.66 times higher risk of developing CP and a 2.09 times higher risk of developing NDI than infants without EUGR_IG_. Although the aOR of the EUGR_F_ for NDI reached statistical significance, it was lower than that of the EUGR_IG_ (1.29 and 2.09, respectively). Furthermore, the DOR of the EUGR_IG_ was higher than that of the EUGR_F_ when CP or NDI were analyzed, with higher DORs indicating better test performance [34]. It could be argued that for predicting neurodevelopmental outcomes such as CP or NDI, the IG-21^ST^ chart is more appropriate than the Fenton chart. Meanwhile, the aOR of growth failure calculated in EUGR_F_ was higher than that of the EUGR_IG_ (2.44 and 1.57, respectively). These were in line with previous studies. Yitayew et al. compared the Fenton and IG-21^ST^ growth charts and found that weight-based EUGR_IG_ may be more strongly associated with poor neurodevelopmental outcomes than EUGR_F_ [13]. Lan et al. reported that the IG-21^ST^ growth chart is not superior to the Fenton chart for assessing preterm growth based on a Chinese retrospective cohort study [35]. Also, recently, Italian researchers compared the Italian Neonatal Study Charts and IG-21^ST^ growth charts and found that EUGR_IG_ could predict neurodevelopmental outcomes in preterm infants more frequently [36].

Due to poor diagnostic effectiveness in terms of sensitivity and/or specificity for both EUGR definitions, it is not a suitable method for predicting long-tern outcomes in clinical practice. The definition of EUGR is primarily based on the physical growth status of infants and is not designed to evaluate neurodevelopment. However, because EUGR is determined through a simple method using easy, noninvasive weight measurements, both EUGR_IG_ and EUGR_F_ could serve as simple indicators useful to clinicians in their everyday practice. When infants are classified as EUGR, neonatologist should be alerted to potential concerns about poor long-term outcomes. Emphasizing early evaluation and intervention for EUGR is crucial when caring for preterm infants.

The strength of our study lies in the utilization of data from a population-based national cohort registry, encompassing approximately 70% of VLBW infants in Korea [14]. The registry maintains a complete data-monitoring system to improve data quality [37]. However, our study is not without limitations. First, we solely focused on weight-based EUGR for the outcome analysis. Second, both the Fenton and IG-21^ST^ charts may not entirely reflect the specific growth patterns of Korean infants due to potential influences from environmental factors and ethnicity.

## 5. Conclusions

In conclusion, this retrospective study investigated the association between long-term growth and neurodevelopmental outcomes and EUGR as defined by either the Fenton or IG-21^ST^ chart in VLBW infants. We found that a higher proportion of CP and NDI at 18–24 months CA was associated with EUGR_IG_ compared to EUGR_F_. Additionally, EUGR_IG_ demonstrated a higher DOR and negative predictive value, suggesting better diagnostic performance in predicting CP or NDI, compared to EUGR_F_. Conversely, EUGR_F_ exhibited a higher DOR and negative predictive value, indicating better diagnostic performance in predicting growth failure, compared to EUGR_IG_. The IG-21^ST^ chart may be more useful for predicting long-term neonatal neurodevelopmental outcomes, and the Fenton chart may be more suitable for predicting growth failure. Meticulous growth monitoring should be consistently implemented throughout the NICU hospitalization of preterm infants. Further studies are needed to investigate the long-term neurodevelopmental outcomes of EUGR preterm infants beyond 24 months of age. Additionally, consideration of the future metabolic risk of overgrowth problems will be helpful in determining the “gold standard” chart for assessing growth in NICU.

## Figures and Tables

**Table 1:** Clinical characteristics of study population according to EUGR.

Characteristics	Fenton			Intergrowth-21		
	EUGR_F_ [*N* = 1606]	Non-EUGR_F_ [*N* = 1043]	^a^ *p*	EUGR_IG_ [*N* = 977]	Non-EUGR_IG_ [*N* = 1672]	^b^ *p*
Maternal Characteristics						
Maternal age, years	33.0 [31.0; 36.0]	33.0 [31.0; 36.0]	0.285	33.0 [31.0; 36.0]	33.0 [31.0; 36.0]	0.831
Maternal HTN	343 (21.4%)	343 (32.9%)	<0.001	168 (17.2%)	518 (31.0%)	<0.001
Cesarian section	1170 (72.9%)	796 (76.3%)	0.052	695 (71.1%)	1271 (76.0%)	0.006
Histological chorioamnionitis	629/1374 (45.8%)	379/950 (39.9%)	0.006	406/824 (49.3%)	602/1500 (40.1%)	<0.001
Antenatal steroid	1352/1581 (85.5%)	870/1027 (84.7%)	0.612	823/959 (85.8%)	1399/1649 (84.8%)	0.534
Infant Characteristics						
Gestational age, weeks	27.9 [26.4; 29.4]	29.1 [27.7; 30.3]	<0.001	27.3 [26.0; 28.7]	29.0 [27.6; 30.1]	<0.001
Birth weight, grams	1030 [850; 1240]	1140 [940; 1300]	<0.01	1000 [840; 1210]	1120 [900; 1300]	< 0.001
5 min AS	7.0 [6.0; 8.0]	7.0 [6.0; 8.0]	0.001	7.0 [6.0; 8.0]	7.0 [6.0; 8.0]	< 0.001
Male sex	815 (50.7%)	548 (52.5%)	0.388	566 (57.9%)	797 (47.7%)	<0.001
SGA_F_	201 (12.5%)	126 (12.1%)	0.786	73 (7.5%)	254 (15.2%)	<0.001
SGA_IG_	273 (17.0%)	193 (18.5%)	0.346	108 (11.1%)	358 (21.4%)	<0.001
Short-term Neonatal Outcomes						
Respiratory distress syndrome	1430 (89.0%)	842 (80.7%)	<0.001	913 (93.4%)	1359 (81.3%)	<0.001
Treated PDA	516/1563 (33.0%)	214/1008 (21.2%)	<0.001	351/952 (36.9%)	379/1619 (23.4%)	<0.001
Moderate to severe BPD	582/1602 (36.3%)	342/1043 (32.8%)	0.068	422/973 (43.4%)	502/1672 (30.0%)	<0.001
Necrotizing enterocolitis	110/1605 (6.9%)	27/1043 (2.6%)	<0.001	86/976 (8.8%)	51/1672 (3.1%)	<0.001
Severe brain injury	108 (6.7%)	39 (3.7%)	<0.001	162 (l6.6%)	122 (7.3%)	<0.001
Retinopathy of prematurity	287/1597 (18.0%)	89/1033 (8.6%)	<0.001	228/971 (23.5%)	148/1659 (8.9%)	<0.001
Sepsis	397 (24.7%)	164 (15.7%)	<0.001	291 (29.8%)	270 (16.1%)	<0.001
Duration of PN, days	27.0 [16.0; 44.0]	17.0 [10.0; 30.5]	<0.001	33.0 [19.0; 51.0]	19.0 [11.0; 31.0]	<0.001
Length of stay, days	77.0 [60.0; 99.0]	63.0 [50.0; 86.0]	<0.001	85.0 [68.0; 107.0]	64.0 [51.0; 84.5]	<0.001

Values are expressed as median (interquartile range [IQR]) or number (percentage). ^a^ *p* values were calculated by comparing EUGR_F_ and non-EUGR_F_ infants and ^b^ *p* values by comparing EUGR_IG_ and non-EUGR_IG_ infants. Abbreviations: AS, Apgar score; BPD, bronchopulmonary dysplasia; EUGR_F_, extrauterine growth restriction defined by Fenton chart; EUGR_IG_, extrauterine growth restriction defined by IG-21^ST^ chart; HTN, hypertension; IG-21^ST^, intergrowth-21st; PDA, patent ductus arteriosus; PN, parenteral nutrition; SGA_F_, small for gestational age by Fenton chart; SGA_IG_, small for gestational age by IG-21^ST^.

**Table 2:** Logistic regression analyses for the relationship between EUGR and long-term outcomes.

**Intergrowth-21**			**Univariate Analysis**			**Multivariate Analysis**		
	**EUGR_IG_**	**Non-EUGR_IG_**	**OR**	**95% CI**	***p***	**aOR**	**95% CI**	***p***
CP ^a^	96/300 (32.0%)	62/390 (15.9%)	2.49	1.73–3.60	<0.001	1.66	1.04–2.65	0.035
NDI ^b^	265/709 (37.4%)	300/1349 (22.2%)	2.09	1.71–2.55	<0.001	2.09	1.71–2.55	<0.001
Growth failure ^c^	158/956 (16.5%)	165/1644 (10.0%)	1.77	1.40–2.24	<0.001	1.57	1.16–2.13	0.004
**Fenton**			**Univariate analysis**			**Multivariate analysis**		
	**EUGR_F_**	**Non-EUGR_F_**	**OR**	**95% CI**	***p***	**aOR**	**95% CI**	***p***
CP ^a^	120/488 (24.6%)	38/202 (18.8%)	1.41	0.94–2.14	0.101	1.30	0.79–2.17	0.310
NDI ^b^	377/1209 (31.2%)	188/849 (22.1%)	1.59	1.30–1.95	<0.001	1.29	1.03–1.63	0.027
Growth failure ^c^	250/1577 (15.9%)	73/1023 (7.1%)	2.45	1.87–3.24	<0.001	2.44	1.77–3.40	<0.001

The ORs and aORs were calculated using non-EUGR_IG_ or non-EUGR_F_ as a reference. aOR is adjusted for the variables that were significantly associated with neonatal outcome in univariate analysis. ^a^ There were 690 infants able to complete evaluation for CP at 18–24 months of corrected age, among them 300 infants classified as EUGR_IG_, and 488 infants classified as EUGR_F_. ^b^ There were 2058 infants able to complete evaluation for NDI at 18–24 months of corrected age, among them 709 infants classified as EUGR_IG_, and 1209 infants classified as EUGR_F_. ^c^ There were 2600 infants able to complete anthropometric measurement at 18–24 months of corrected age, among them 956 infants classified as EUGR_IG_, and 1577 infants classified as EUGR_F_. Abbreviations: aOR, adjusted odds ratio; CI, confidence interval; CP, cerebral palsy; EUGR_F_, extrauterine growth restriction defined by Fenton chart; EUGR_IG_, extrauterine growth restriction defined by IG-21^ST^ chart; IG-21^ST^, intergrowth-21st; OR odds ratio; NDI, neurodevelopmental impairment.

**Table 3:** Diagnostic effectiveness for long-term outcomes.

	EUGR_IG_ (95% CI)	EUGR_F_ (95% CI)
CP		
Sensitivity	60.76 (52.69–68.42)	75.95 (68.52–82.38)
Specificity	52.78 (47.95–57.57)	30.83 (26.92–34.94)
Positive predictive value	32.00 (28.62–35.58)	24.59 (22.70–26.58)
Negative predictive value	78.62 (74.81–81.99)	81.19 (76.09–85.41)
Accuracy	54.92 (50.8–58.98)	41.16 (37.46–44.94)
DOR	1.73 (1.19–2.50)	1.40 (0.93–2.11)
NDI		
Sensitivity	46.90 (42.72–51.11)	66.73 (62.67–70.60)
Specificity	70.26 (67.87–72.57)	44.27 (41.73–46.84)
Positive predictive value	37.38 (34.67–40.16)	31.18 (29.62–32.79)
Negative predictive value	77.76 (76.27–79.18)	77.86 (75.54–80.01)
Accuracy	63.85 (61.73–65.93)	50.44 (48.25–52.62)
DOR	2.08 (1.71–2.54)	1.59 (1.30–1.95)
Growth Failure		
Sensitivity	48.92 (43.34–54.51)	77.40 (72.44–81.85)
Specificity	64.95 (62.95–66.92)	41.85 (39.81–43.91)
Positive predictive value	16.53 (14.88–18.32)	15.92 (15.03–16.86)
Negative predictive value	89.96 (88.92–90.92)	92.86 (91.36–94.12)
Accuracy	62.96 (61.07–64.82)	46.28 (44.35–48.22)
DOR	1.77 (1.40–2.24)	2.46 (1.87–3.24)

Abbreviations: CI, confidence interval; CP, cerebral palsy; DOR, diagnostic odds ratio; EUGR_F_, extrauterine growth restriction defined by Fenton chart; EUGR_IG_, extrauterine growth restriction defined by IG-21^ST^ chart; IG-21^ST^, intergrowth-21st; NDI, neurodevelopmental impairment.

## Data Availability

The data that support the findings of this study are available from the KNN, but restrictions apply to the availability of these data, which were used under license for the current study and are not publicly available.

## Appendix A

**Table A1:** Logistic regression analysis model for the relationship between neonatal risk factors and long-term outcome.

	CP			NDI			Growth Failure		
	OR	95% CI	*p* Value	OR	95% CI	*p* Value	OR	95% CI	*p* Value
Maternal characteristics									
Maternal age, years	1.02	0.98–1.07	0.312	1.00	0.98–1.03	0.782	1.00	0.97–1.02	0.812
Maternal HTN	0.70	0.44–1.08	0.112	1.11	0.89–1.38	0.354	1.93	1.51–2.46	<0.001
C/S	1.19	0.80–1.80	0.398	1.09	0.87–1.37	0.455	2.41	1.75–3.40	<0.001
HCAM	1.08	0.73–1.59	0.701	0.87	0.71–1.07	0.192	0.74	0.57–0.95	0.019
Antenatal steroid	0.80	0.51–1.27	0.328	0.92	0.70–1.21	0.540	1.21	0.86–1.74	0.291
Infantile characteristics									
GA, weeks	0.81	0.74–0.89	<0.001	0.83	0.79–0.88	<0.001	0.94	0.88–1.00	0.037
BW, grams	1.00	1.00–1.00	0.008	1.00	1.00–1.00	<0.001	1.00	1.00–1.00	<0.001
5 min AS	0.93	0.84–1.03	0.140	0.87	0.83–0.92	<0.001	0.89	0.83–0.95	<0.001
Male.sex	1.01	0.71–1.45	0.939	1.49	1.23–1.82	<0.001	1.18	0.94–1.50	0.157
Short-term Neonatal Outcomes									
RDS	1.89	1.01–3.87	0.060	1.75	1.30–2.40	<0.001	1.28	0.90–1.85	0.182
Treated PDA	1.47	1.01–2.13	0.044	1.49	1.21–1.84	<0.001	1.67	1.30–2.13	<0.001
Moderate to severe BPD	1.90	1.32–2.72	<0.001	2.28	1.87–2.78	<0.001	2.57	2.03–3.26	<0.001
NEC	1.70	0.87–3.18	0.108	1.71	1.16–2.51	0.006	1.58	0.98–2.46	0.050
Severe brain injury	12.59	8.17–19.68	<0.001	4.10	3.07–5.50	<0.001	1.98	1.43–2.71	<0.001
ROP	12.59	8.17–19.68	<0.001	2.04	1.58–2.63	<0.001	2.54	1.92–3.33	<0.001
Sepsis	1.22	0.79–1.83	0.360	1.62	1.29–2.03	<0.001	1.37	1.04–1.78	0.022
Duration of PN, days	1.01	1.00–1.02	0.002	1.02	1.01–1.02	<0.001	1.02	1.01–1.02	<0.001
Length of stay, days	1.02	1.02–1.03	<0.001	1.02	1.01–1.02	<0.001	1.02	1.02–1.03	<0.001
EUGR_F_	1.41	0.94–2.14	0.101	1.59	1.30–1.95	<0.001	2.45	1.87–3.24	<0.001
EUGR_IG_	2.49	1.73–3.60	<0.001	2.09	1.71–2.55	<0.001	1.77	1.40–2.24	<0.001

EUGR was defined as decreased z scores of weight >1 at between birth and discharge: referred to as EUGR_IG_ using IG-21^ST^ chart and as EUGR_F_ using Fenton chart. Abbreviations: AS, Apgar score; BPD, bronchopulmonary dysplasia; BW, birth weight; C/S, cesarian section; EUGR, extrauterine growth restriction; GA, gestational age; HTN, hypertension; HCAM, histological chorioamnionitis; NEC, necrotizing enterocolitis; PDA, patent ductus arteriosus; PN, parenteral nutrition; RDS, respiratory distress syndrome; ROP, retinopathy of prematurity, SGA, small for gestational age.
